# Clinical and Genetic Characterisation of Cystic Fibrosis Patients in Latvia: A Twenty-Five-Year Experience

**DOI:** 10.3390/diagnostics12112893

**Published:** 2022-11-21

**Authors:** Madara Auzenbaha, Elina Aleksejeva, Gita Taurina, Liene Kornejeva, Inga Kempa, Vija Svabe, Linda Gailite

**Affiliations:** 1 Scientific Laboratory of Molecular Genetics, Riga Stradins University, LV-1007 Riga, Latvia; 2 Pulmonology and Allergology Department, Children’s Clinical University Hospital, LV-1004 Riga, Latvia; 3 Riga Maternity Hospital, LV-1013 Riga, Latvia

**Keywords:** cystic fibrosis, CFTR, novel variants

## Abstract

Cystic fibrosis (CF) is the most common life-limiting genetic disorder in European descent populations. It is caused by pathogenic variants in the *CFTR* gene, and inheritance is autosomal recessive. This study provides an up-to-date, comprehensive estimation of the distribution of *CFTR* pathogenic variants in Latvia and their phenotypic characteristics. It also reports the first results of the CF newborn screening programme following its implementation in 2019. We analysed the clinical and molecular data of CF patients treated at the only tertiary hospital in Latvia providing specialised healthcare for the disorder. Between 1997 and 2022, 66 CF patients from 62 families were diagnosed based on symptoms or a molecular confirmation (six patients were diagnosed through the CF newborn screening programme). F508del was identified in 70.5% of all CF chromosomes. Known variants were identified in more than one family: dele2,3, R1006H, L1335P, W57R, R553X, 2143delT and 3849+10kb C>T (legacy names used). Furthermore, two novel variants were identified, namely, c.503C>A p.(Ser168Ter) and c.(743+1_744-1)_(1584+1_1585-1)del p.(?). The available follow-up results indicated that Latvian CF patients demonstrated similar tendencies to CF patients worldwide. The oldest age at diagnosis prior to the implementation of the CF newborn screening programme was 14 years. We provide here, for the first time, a comprehensive description of Latvian CF patients. An improvement in the healthcare of CF patients over time, including access to diagnosis, is evident. Two novel CF-causing variants are reported, and F508del is the most frequently occurring variant in the population, thus suggesting that F508del screening should be followed by the testing of the full *CFTR* gene.

## 1. Introduction

Cystic fibrosis (CF; MIM #219700) has an autosomal recessive mode of inheritance and is the most common life-limiting genetic disorder in Caucasian populations [1]. In Europe, the incidence ranges from 1/1353 in Ireland to 1/25,000 in Finland and is, on average, 1/4500 in Western Europe and 1/6000 in Northern and Central Europe [2]. The Cystic Fibrosis Foundation reports that 60,000 to 70,000 people worldwide suffer from CF [3].

CF is caused by pathogenic variants/mutations in the *cystic fibrosis transmembrane conductance regulator* (*CFTR*) gene and is characterised by a multiorgan pathology affecting the upper and lower airways, gastrointestinal and reproductive tracts and endocrine system [4].

To date, more than 2000 *CFTR* variants have been identified [5]. Central, Northern, Western and Northeastern Europe show a large degree of homogeneity among the *CFTR* mutations. On average, 10.2 mutations per country account for 78.9% of the total number of CF chromosomes in these regions. This is largely attributable to the high prevalence of the F508del variant in these regions [6]. This variant is also the most frequent one in the general Caucasian population, accounting for ∼66% of CF chromosomes. Only a few *CFTR* variants reach worldwide frequencies of >1%, fewer than 20 have frequencies between 0.1 and 1.0% and the majority are found only in certain geographical regions, populations or single families (termed ‘private’) [7].

Currently, six major classes of *CFTR* mutations are distinguished. Class I mutations result in severely reduced or absent *CFTR* expression. Class II mutations severely reduce the number of CFTR molecules that reach the cell surface. Class III mutations impair the regulation of the CFTR channel, resulting in abnormal gating characterised by a reduced open probability. Class IV mutations alter the channel conductance by impeding the ion conduction pore, leading to a reduced unitary conductance. Class V mutations do not change the conformation of the protein but alter its abundance by introducing promoter or splicing abnormalities. Class VI mutations destabilise the channel in post-endoplasmic reticulum compartments and/or at the plasma membrane [4]. As studies have shown that a genetic variant can have characteristics of multiple classes, understanding the functionality of each mutation class is crucial for the development of drug combination strategies to improve the available treatments for CF patients [4]. Mutation classification is important as specific treatments are created, and that is the reason why some authors are suggesting to export variants in which no mRNA is created from the Class I into the new Class VII and to rename the mutation class to Theratype [8,9,10]. As guidelines in reclassification are still missing, we used the existing classification.

Presently, nationwide CF newborn screening is offered in nearly all European countries, the United States of America, Canada, Australia and even Russia, Turkey and Brazil [11]. In Latvia, nationwide CF newborn screening was implemented in 2019, following a 2009 pilot study [12]. Prior to the CF newborn screening programme, diagnosis was made after clinical manifestation (confirmed with a positive sweat test (chloride concentration > 60 mmol/L)) or through family anamnesis.

This study provides an up-to-date comprehensive estimation of the distribution of *CFTR* pathogenic variants in Latvia and their phenotypic characteristics. It also reports the first results of the CF newborn screening programme following its implementation in 2019.

## 2. Materials and Methods

Written informed consent was obtained from all individuals involved in the study. The study was performed according to the Declaration of Helsinki, and the protocol was approved by the Central Medical Ethics Committee of Latvia.

A clinical diagnosis of CF was established in 62 unrelated symptomatic patients (4 siblings) and 6 pre-symptomatic patients (through newborn screening) according to clinical and laboratory diagnostic consensus criteria [13]—in total, 70 patients. They represented all the CF patients in Latvia during the 25-year period from 1997 to June 2022. Due to various reasons (e.g., death (*n* = 18), emigration), only 45 patients are currently under clinical follow-up.

Over the 25-year period, our method for the molecular analysis of *CFTR* (HGNC:1884, reference sequences: NG_016465.4, NM_000492.3, LRG_663) evolved as new technologies were developed. The initial mutation screen was performed by polymerase chain reaction (PCR) for c.1521_1523delCTT p.(Phe508del) (legacy name F508del variant) and 54-5940_273+10250del21kb p.(Ser18ArgfsX16) (legacy name CFTRdele2,3) using duplex PCR and allele-specific PCR adapted from previous studies. Subsequently, PCR with restriction fragment length polymorphism (1998–2005), single-stranded conformation polymorphism with Sanger sequencing (2005–2007), APEX (2007–2012) and Elucigene (2012–2015) were the technologies employed. Since 2015, we have sequenced the whole coding part of *CFTR* using ThermoFisher’s BigDye Terminator v.3.1 Cycle Sequencing Kit (according to their protocol) and previously published primers [14]. In addition to the coding part, our current analysis also includes two deep intronic variants (c.3718-2477C>T p.(?) (legacy name 3849+10kbC>T) and c.1680-877G>T p.(?) (legacy name 1811+1.6kbA>G)), followed by multiplex ligation-dependent probe amplification (MRC-Holland). If two CF-causing variants were identified, to confirm variant localisation in the *trans* position and thus CF diagnosis, the parents were tested accordingly.

The databases CFTR1, CFTR2 and ClinVar were used for clinical interpretation. For rare variants, the American College of Medical Genetics guidelines were used.

The CF newborn screening strategy was as follows: immunoreactive trypsinogen (IRT) measured by a fluorometric enzyme immunoassay and Fluoroskan Ascent microplate fluorometer. If >70 ng/mL, then a second IRT measurement was performed. If elevated again, then DNA analysis (method detailed above) and a sweat test were performed in parallel. The IRT cut-off was set at 50 ng/mL for the first three months, after which it was adjusted to 70 ng/mL.

## 3. Results

### 3.1. CF Screening Results

A total of 50,859 samples were subjected to IRT assessment. The number of children screened represented more than 99% of the children born in the period. In total, 772 (1.5%) were re-assessed for IRT, with 184 having an increased IRT for the second time. Consequently, these 184 children underwent DNA analysis and a sweat test. Seven infants with two pathogenic *CFTR* variants were reported for diagnostic follow-up, giving an incidence of 1:7265.

### 3.2. CFTR Genotype

A total of 21 pathogenic variants in the *CFTR* gene were found in 128 of 132 (97%) affected chromosomes in the Latvian population. Two of these variants, c.503C>A p.(Ser168Ter) in exon 5 and c.(743+1_744-1)_(1584+1_1585-1)del p.(?) covering exon 7 through to exon 11, have not been previously described. Table 1 shows the frequency and pathogenicity class of the 21 *CFTR* CF-causing variants found in our patients. In total, 13.1% of Latvian patients carried Class I mutations, and 75.1% carried at least one Class II mutation.

Of 66 CF individuals, two pathogenic or likely pathogenic variants were found in 62. A second pathogenic variant could not be identified in four cases (3% of alleles). This was because our current extensive molecular analysis of *CFTR* was not implemented until 2015, the patients were already deceased and additional DNA material was not available (Table S1 summarises the genotype results). The frequencies of the variants identified in our Latvian patients were compared with those reported in our closest neighbours to establish the degree of similarity (Table 2).

In 13 of the 70 (18.6%) Latvian patients, we revealed rare *CFTR* genotypes; their compound heterozygous state was confirmed by parental testing. Some of these rare cases that were available for follow-up are briefly phenotypically presented below.

#### 3.2.1. Case 1 with Genotype c.[1521_1523delCTT];[169T>C] p.[(Phe508del)];[(Trp57Arg)], Legacy Nomenclature dF508/W57R

He was a male patient. CF diagnosis was confirmed at 10 months of age in 1993 by a positive sweat test result of 200 mmol/L. Poor weight gain, fatty stools, wet cough and symptoms of gastro-oesophageal reflux disease (GORD) were present from the first months of life. From the age of 6 years, he had severe pulmonary exacerbations (PExs) at least four times a year, which were treated with intravenous antibiotics. He developed chronic rhinosinusitis at the age of 8. Chronic multiresistant *Pseudomonas aeruginosa* infection was recognised early at the age of 12. At the age of 14, his disease was complicated by renal amyloidosis and progressive renal failure. He died at the age of 15 due to progressive respiratory failure.

#### 3.2.2. Case 2 with Genotype c.[3844T>C];[2012delT] p.[(Trp1282Arg)];[(Leu671Ter)], Legacy Nomenclature W1282R/2143delT

She was a female patient. CF diagnosis was assumed at 4 months of age due to the clinical presentation of pancreatic exocrine insufficiency and productive cough and was subsequently confirmed with a positive sweat test result of 126 mmol/L. Despite receiving regular pancreatic enzyme replacement therapy with pancreatin, she has problems with weight and height gain. Accordingly, since the age of 9 years, a percutaneous endoscopic gastrostomy probe has provided enteral feeding. From the age of 4, she has had the frequent recurrence of PExs; seven episodes in the last year required hospitalisation and intravenous antibiotics. The patient is currently 10 years old.

#### 3.2.3. Cases 3 and 4 with Genotype c.[1521_1523delCTT];[3844T>C] p.[(Phe508del)];[(Trp1282Arg)], Legacy Nomenclature dF508/W1282R

**Figure 1:**
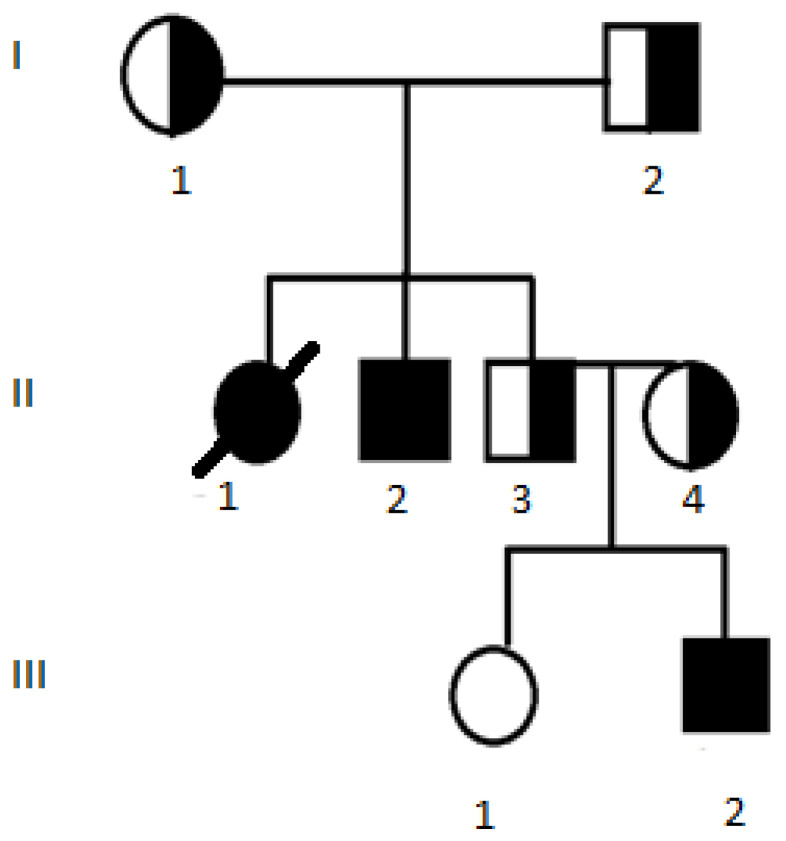
Pedigree for Case 3 (II-1) and Case 4 (II-2). III-2 has genotype c.[1521_1523delCTT];[1521_1523delCTT] p.[(Phe508del)];[(Phe508del)], legacy nomenclature dF508/dF508.

These cases involve two siblings—female and male (shown in Figure 1). The CF diagnosis of II-1 was confirmed by a positive sweat test result of 136 mmol/L at the age of 3 years. Symptoms of malabsorption with poor weight gain, fatty stools from birth and early respiratory symptoms (persistent and productive cough) were observed. GORD symptoms were observed periodically since adolescence. She had CF-associated diabetes since the age of 24. She died at the age of 32, with PExs escalating to seven episodes requiring hospitalisation in her final year, the progression of respiratory failure and the development of secondary pulmonary hypertension.

Her brother, II-2, did not have his CF diagnosis confirmed until the age of 24 years. This was because his parents would not consent to him nor his healthy brother being assessed earlier. However, their attitude changed after the healthy brother’s second child was diagnosed with CF shortly after birth (III-2). Patient II-2 has mild pancreatic insufficiency and respiratory symptoms, despite chronic *Pseudomonas aeruginosa* colonisation, with mild PExs. The patient is currently 30 years old.

Patient III-2 is a male relative of the two previous cases. Due to the family history of CF, he was molecularly diagnosed with CF—homozygous for c.1521_1523delCTT p.(Phe508del)—shortly after birth. Concurrently, his diagnosis was clinically confirmed by a positive sweat test result of 122 mmol/L. He has had symptoms of pancreatic insufficiency since birth and a productive cough from 6 months of age. He also has the chronic colonisation of *Staphylococcus aureus* in his airways and mild PExs twice a year, which are treated with oral antibiotics. His episodic symptoms of GORD are treated with proton pump inhibitors. The patient is currently 6 years old. Interestingly, this pedigree shows that, in some families, a rare disease may occur more frequently than estimated.

#### 3.2.4. Cases 5 and 6 with Genotype c.[1521_1523delCTT];[(743+1_744-1)_(1584+1_1585-1)dup] p.[(Phe508del)];[(?)], Legacy Nomenclature dF508/dup6b-10

These cases involve two male siblings. CF diagnosis of the index case was established at the age of 3 years. The diagnosis was confirmed by a positive sweat test result of 97 mmol/L. The disease at the time of diagnosis was manifested by respiratory tract (wet cough) and pancreatic insufficiency (poor weight gain, fatty stools) symptoms. GORD, oesophagitis and CF liver disease were diagnosed at the age of 12. Over a 25-year follow-up period, the patient has had mild PExs once or twice a year, which are most often treated with oral antibiotics; although, for the last two years, he has received one course of intravenous antibiotics each year. He has chronic *Staphylococcus aureus* colonisation in his airways; *Haemophilus influenzae* infection was detected once and successfully treated with one eradication course. The patient is currently 28 years old.

His sibling was clinically diagnosed with CF shortly after birth. The diagnosis was confirmed by a positive sweat test result of 100 mmol/L. At the time of diagnosis, fatty stools were observed, and, consequently, pancreatic enzyme replacement therapy was started. Respiratory symptoms appeared at the age of 1 year. The patient has mild PExs a couple times a year, which are treated with oral antibiotics. A diagnosis of CF liver disease was confirmed at the age of 14. He has chronic *Staphylococcus aureus* colonisation in his airways. The patient is currently 19 years old.

#### 3.2.5. Case 7 with Genotype c.[503C>A];[4004T>C] p.[(Ser168Ter)];[(Leu1335Pro)], Legacy Nomenclature Ser168Ter/L1335P

She is a female patient. CF diagnosis was made at 3 weeks of age through the CF newborn screening programme (IRT 121.13 µg/L on day 8, IRT 83.16 µg/L on day 16) and was confirmed with two positive sweat tests (84 and 85 mmol/L). The patient is pancreatic sufficient; elastase 300 µg/kg. During her first year, she had symptoms of GORD (treated with proton pump inhibitors), recurrent episodes of wheezing bronchitis and the chronic colonisation of *Staphylococcus aureus* and *Haemophilus influenzae*. In her second year, she has had mild PExs twice (treated with oral antibiotics). The patient is currently 1 year and 11 months old.

### 3.3. Clinical and Laboratory Characteristics of CF Patients under Regular Follow-Up

From a total of 66 patients (62 index patients), regular follow-up data were available for only 45 cases (Table 3). During 2021, two patients died and nine ceased to be followed up. 

## 4. Discussion

This study presents a comprehensive overview of Latvian CF patients by investigating a representative cohort of 66 patients (62 unrelated index cases) over a 25-year period. Two-and-a-half years of newborn screening revealed a lower incidence of CF than previous calculations based on F508del carrier detection in 2001 and a CF newborn screening pilot study [12]. However, other studies have reported similar data indicating that epidemiological changes have occurred both in the incidence of CF, which appears to be decreasing in most countries, and in the survival of CF patients, which has greatly improved in recent decades [2]. For instance, in the Slovak population, the CF incidence was first estimated in 1979 at a frequency of 1:1800 newborns; however, three decades later, neonatal genetic screening between 2009 and 2015 revealed a lower incidence of 1:6315–7668 [25].

In Europe, countries such as Norway and the United Kingdom have offered newborn screening for CF for many years, whereas our neighbouring countries, Estonia and Lithuania, have yet to implement CF newborn screening [26]. Therefore, it is difficult at present to compare our data with those from geographically close populations. As we implemented our CF newborn screening programme only very recently (1 August 2019), it is still being optimised. One concern is the possibility of false negative cases as, fewer new cases than expected have been diagnosed since we started to screen newborns for CF. In Latvia, we have adopted the protocol of two successive elevated IRT measurements, followed by a sweat test and molecular analysis of the whole *CFTR* gene. However, in the near future, we plan to improve this protocol with earlier *CFTR* molecular analysis (i.e., IRT/DNA/IRT), similar to the procedures employed in Poland [27] and Switzerland [28]. After CF NBS implementation, six patients are discovered. Two had meconium ileus, which would lead to an early CF diagnosis anyway, but four cases, including a case with two rare mutations, were possible to diagnose so early only because CF NBS, as significant symptoms, were not present at the moment. Five out of six patients have the most common genotype identified: homozygoys F508del variant. Clinical symptoms comparison with patients diagnosed before and during the screening for the patients with the same genotype was not performed because the patients are still young, and long-term prognosis could only be predicted, but also has been changed used treatment. 

A 2014 study reported that Latvia had a high frequency of *CFTR* Class II mutations (>90%) in comparison with other European countries; however, the study sample consisted of only 29 CF patients [29]. The present study, with a higher patient count, found that 75.1% of CF patients carried at least one *CFTR* Class II mutation. We identified very few patients with Class III mutations, which are interpreted differently by various researchers [4, 18]. In contrast, the frequency of Class III mutations has been reported to be 13.9% and 6.5% in Ireland and the United Kingdom, respectively [29]. We identified the F508del variant in 70.5% of all CF chromosomes. This is the highest frequency among the Baltic countries, with 52% and 51.7% being reported in Lithuania [16] and Estonia [20], respectively. However, it should be noted that these frequencies were reported over 15 years ago. Furthermore, in small populations, such as the one in Latvia, even just a few patients can change the data significantly. Molecular analysis of our patients’ genotypes revealed that 37 out of 45 were eligible for CFTR modulating therapy with elexacaftor/tezacaftor/ivacaftor and ivacaftor. This treatment is not yet available in Latvia but will hopefully be accessible soon. Four (6%) of our patients had a genotype for which CFTR modulating therapy is not suggested. It has recently been reported that approximately 10% of CF patients are not eligible for this type of therapy [30]. Hence, the development of effective therapies for these patients is a priority.

We identified two novel *CFTR* variants in our patient cohort. One patient (Case 7; pancreatic sufficient) carried a rare variant and a novel variant, c.503C>A p.(Ser168Ter). This patient was diagnosed through the CF newborn screening programme and exemplifies why it is necessary to conduct a full scan of *CFTR* after F508del analysis. Her novel point variant is interpreted as CF-causing based on the following American College of Medical Genetics criteria [31]: PVS1—the variant leads to a premature STOP codon, and protein function loss is a known mechanism for the development of the disease; PS1—the variant c.503C>T p.(Ser168Leu) has previously been described in the literature and identified in CF patients [32]; PM2—the variant is absent from the Genome Aggregation Database (gnomAD); PM3—the variant is in the *trans* position with a known CF-causing variant; PP4—CF newborn screening of the patient reveals two successive elevated IRT measurements and two positive sweat tests.

Interestingly, almost all of our molecular findings are shared with native Russians. For example, a very rare variant, c.3844T>C p.(Trp1282Arg) [23], that is not even reported in *CFTR* databases was detected at a higher incidence in our CF patients than in the Russian population (1.5% versus 0.76%). Researchers in Russia have classified it as a Class II or III variant, showing pancreatic insufficiency and a significant decrease in lung function. Indeed, both of our patients with this variant in the compound heterozygous state (F508del/W1282R and W1282R/2143delT) showed pancreatic insufficiency and a severe disease course. We were unable to find a mention of the W1282R/2143delT combination of *CFTR* variants in the literature, although Petrova et al. have reported the frequency of the 2143delT variant in Russia—2.71% [17]. Nevertheless, our patient with this *CFTR* variant combination displayed a classic CF phenotype with pancreatic insufficiency. To the best of our knowledge, both of our patients with the W1282R variant are of Latvian origin. Thus, this variant could be characteristic of Latvians as well; however, future studies focusing on native Latvians need to be conducted to confirm this. As mentioned above, we plan, in the near future, to change our newborn screening algorithm and perform DNA analysis directly after the measurement of the first elevated level of IRT. Our first-line DNA analysis will include W1282R as well as c.1545_1546delTA p.(Tyr515Ter) and c.169T>C p.(Trp57Arg), described in the Finnish population [22]. These variants are not included in the first-line panel of our neighbouring countries, such as Poland [27]. As it is evident that *CFTR* variants are highly heterogeneous among different populations, we believe that local studies and registries are very important, even in countries with relatively small numbers of CF patients.

Latvia has a relatively small population of approximately two million, consequently giving rise to a proportionally small number of CF patients. The available budget for rare disease treatment in Latvia is not as extensive as that in other European countries. For example, when dornase alfa was approved as a treatment for CF, only some of our patients received it due to financial constraints. Similarly, the only CFTR modulator available in Latvia is lumacaftor/ivacaftor (since the beginning of 2022). Furthermore, lung transplantation is not currently a realistic option, as Latvia is not yet a member of Scandiatransplant (http://www.scandiatransplant.org/ (accessed on 1 November 2022)). Therefore, transplantation would only be possible if the donor was Latvian and the patient’s clinical status allowed for it. Taking all these factors into consideration, the clinical data we have presented here reflect a good quality of care for CF patients in Latvia.

Regular follow-up is an important part of CF patient management. Over the last 10 years, death (in 2021, two patients died at the ages of 24 and 35 years) and emigration have resulted in patients not being followed up. Additionally, two patients attend follow-up irregularly, correlating with their adherence to treatment plans. It has been identified that a patient’s adherence to their treatment plan is stronger at an earlier age and decreases as patients grow up [33]. Parental depression has been proposed as a factor in treatment adherence based on the findings of a study conducted in Ireland [34]. In the present study, parental refusal to test offspring resulted in a delay in the molecular diagnosis of one of our patients described in the family of Cases 3 and 4.

Our patients showed a relatively low *Pseudomonas aeruginosa* infection rate (22.2%) in comparison to the colonisation rate reported by Petrova et al. in Bulgarian CF patients (60%) [21]. In Cypriot CF patients, 53.9% were found to be positive for the *Pseudomonas aeruginosa* pathogen, and 26.9% had chronic *Pseudomonas aeruginosa* colonisation [35]. 

A study of longitudinal changes in nutrition using BMI values over a 10-year period has reported that underweight CF patients decreased from 20.6% to 11.1%, while overweight/obese CF patients increased from 7% to 18.4% [36]. Most of our patients were found to be in the normal weight group, where the BMI is between 19 and 25, which is very important for better lung function.

## 5. Conclusions

The present study provides a comprehensive description of Latvian CF patients by documenting the genetic background of *CFTR* in the Latvian population and reporting two novel variants. Furthermore, the first results of the Latvian CF newborn screening programme since it was implemented in 2019 are presented. The challenges faced by countries like Latvia with relatively small populations, such as a modest healthcare budget and limited clinical experience, are also discussed. Lastly, due to the Latvian population’s unique ethnicity, several notable differences attributable to regional peculiarities are highlighted.

## Figures and Tables

**Table 1:** Frequency and pathogenicity class of *CFTR* CF-causing variants found in 66 unrelated Latvian patients.

Allele According to Legacy Nomenclature	Exon/ Intron NM_000492.3	HGVS Nomenclature	Class of *CFTR* Mutation	N	% of CF-Causing Alleles in Latvia
W57R	3	c.169T>C p.(Trp57Arg)	II or III ^a^	2	1.5%
394delTT	3	c.262_263delTT p.(Leu88IlefsX22)	I [15]	1	0.8%
P67L	3	c.200C>T p.(Pro67Leu)	II-III [4]	1	0.8%
L138ins	4	c.413_415dupTAC p.(Leu138dup)	II ^b^	1	0.8%
621+1G>T	4	c.489+1G>T p.(?)	I [15]	1	0.8%
Ser168Ter	5	c.503C>A p.(Ser168Ter)	I ^c^	1	0.8%
R334W	8	c.1001G>A p.(Arg334Gln)	III [15], II-III [4]	1	0.8%
A455E	10	c.1364C>A p.(Ala455Glu)	II [15], II-III [4]	1	0.8%
F508del	11	c.1521_1523delCTT p.(Phe508del)	II-III [15], II-III-VI [4]	93	70.5%
1677delTA	11	c.1545_1546delTA p.(Tyr515X)	I ^c^	2	1.5%
R553X	11	c.1657C>T p.(Arg553X)	I ^c^	2	1.5%
2143delT	14	c.2012delT p.(Leu671Ter)	I ^c^	2	1.5%
2184insA	14	c.2052dupA p.(Gln685ThrfsX5)	I ^c^	1	0.8%
R1006H	20	c.3197G>A p.(Arg1066His)	II-III [4]	3	2.3%
W1282X	23	c.3846G>A p.(Trp1282Ter)	I-II-III-VI [4]	2	1.5%
W1282R	23	c.3844T>C p.(Trp1282Arg)	II-III [16,17]	2	1.5%
L1335P	25	c.4004T>C p.(Leu1335Pro)	II ^d^	3	2.3%
dele2,3	covering 2nd and 3rd exons	c.54-5940_273+10250del21kb p.(Ser18ArgfsX16)	I [15]	5	3.8%
CFTRdup6b-10	covering 7th–11th exons	c.(743+1_744-1)_(1584+1_1585-1)dup p.(?)	I ^c^	1	0.8%
CFTRdel6b-10	covering 7th–11th exons	c.(743+1_744-1)_(1584+1_1585-1)del p.(?)	I ^c^	1	0.8%
3849+10kb C>T	intron between 22nd and 23rd exons	c.3718-2477C>T p.(?)	V [15]	2	1.5%
not identified				4	3.0%

^a^ Classified as Class II or III, as there are no published functional studies and the variant is not from Class I and is clearly a CF-causing allele. ^b^ Classified as Class II, as a drug efficiency experiment classified the variant as a high responder to lumacaftor (a folding corrector) and as having 1.6% residual activity [18]. ^c^ All frameshift, nonsense and large deletions/duplications are classified as Class I according to the general description [15]. ^d^ Classified as Class II or III, as published functional studies state that this variant retains 2.6% of wildtype activity and is clearly CF-causing [19].

**Table 2:** Frequencies of *CFTR* variants detected in CF patients from Latvia and neighbouring countries.

Variant Legacy Name.	Variant HGVS Nomenclature	Latvia %	Estonia ^a^ %	Lithuania ^b^ %	Poland ^c^ %	Bulgaria ^d^ %	Finland ^e^ %	Russia (Ethnic) ^f^ %	Jewish ^g^ %
F508del	c.1521_1523delCTT p.(Phe508del)	70.5	51.7	52	54.54	55	36	54.99	35.6
dele2,3	c.54-5940_273+10250del21kb p.(Ser18ArgfsX16)	3.8	0	2	4.47	0	5.9	7.59	0
R1006H	c.3197G>A p.(Arg1066His)	2.3	0.017	0	0	0	0	0	0
L1335P	c.4004T>C p.(Leu1335Pro)	2.3	0	0	0	0.36	0	0.22	0
W57R	c.169T>C p.(Trp57Arg)	1.5	0	0	0	0	1	0	0
1677delTA	c.1545_1546delTA p.(Tyr515X)	1.5	0	0	0	1.07	0	0.18	0
R553X	c.1657C>T p.(Arg553X)	1.5	0	4.2	0	0	0	0.18	0
2143delT	c.2012delT p.(Leu671Ter)	1.5	0	0	0	0	0	2.71	0
W1282X	c.3846G>A p.(Trp1282Ter)	1.5	0	1	0.61	1.43	0	1.16	31.3
W1282R	c.3844T>C p.(Trp1282Arg)	1.5	0	0	0	0	0	0.76	0
3849+10kb C>T	c.3718-2477C>T p.(?)	1.5	0	0	3.93	1.43	0	2.35	4.6
394delTT	c.262_263delTT p.(Leu88IlefsX22)	0.8	13.3	0	0	0	35	0.54	0
P67L	c.200C>T p.(Pro67Leu)	0.8	0	0	0	0	0	0	0
L138ins	c.413_415dupTAC p.(Leu138dup)	0.8	0	0	0	0	0	1.12	0
621+1G>T	c.489+1G>T p.(?)	0.8	0	0	0.34	1.43	0	0.25	0
Ser168Ter	c.503C>A p.(Ser168Ter)	0.8	0	0	0	0	0	0	0
R334W	c.1001G>A p.(Arg334Gln)	0.8	0	0	0.41	0.71	0	0.69	0
A455E	c.1364C>A p.(Ala455Glu)	0.8	0	0	0	0	0	0.04	0.4
2184insA	c.2052dupA p.(Gln685ThrfsX5)	0.8	0	0	1.02	2.89	0	2.24	0
CFTRdup6b-10	c.(743+1_744-1)_(1584+1_1585-1)dup p.(?)	0.8	0	0	0	0	0	0.18	0
CFTRdel6b-10	c.(743+1_744-1)_(1584+1_1585-1)del p.(?)	0.8	0	0	0	0	0	0	0
not identified		3.0	13.3	35.8	17.5	1.07	2.9	17.22	2.5
No. of alleles analysed		132	60	98	1476	280	102	2768	281

^a^ Estonia [20]; ^b^ Lithuania [16]; ^c^ Poland [7]; ^d^ Bulgaria [21]; ^e^ Finland [22]; ^f^ Russia (ethnic) [23]; ^g^ Jewish [24].

**Table 3:** Characterisation of CF patients currently under regular follow-up in 2022.

Characteristic	Value
Number of patients	45
Male/female ratio	19/26
Mean age at follow-up (years) ± SD (min–max)	13.2 ± 12.2 (1.4–35)
Mean age at diagnosis (years) (min–max)	2.58 (0–14)
Number of patients older than 18 years (%)	13 (28.9%)
Mean Z-score for BMI aged 2–17 years (min–max)	−0.4 (−2.3–1.4)
Mean BMI aged 18 years or older ± SD (min–max)	19.0 (15.6–23.9)
Mean FEV1 (% predicted) ± SD (min–max)	79.7 (18.3–111.9)
Prevalence of *Pseudomonas aeruginosa* infection (%)	10 (22.2%)
Prevalence of *Burkholderia* species infection (%)	1 (2.2%)
Prevalence of *Staphylococcus aureus* infection (%)	28 (62.2%)
Prevalence of methicillin-resistant *Staphylococcus aureus* infection (%)	1 (2.2%)
Prevalence of non-tuberculous *Mycobacterium* species infection	0
Prevalence of *Stenotrophomonas maltophilia* infection (%)	3 (6.7%)
Prevalence of *Haemophilus influenzae* infection (%)	3 (6.7%)
Prevalence of *Achromobacter* species infection (%)	4 (8.9%)
CF liver disease (%)	14 (31.1%)
CF-related diabetes mellitus (%)	3 (6.7%)
Pancreatic sufficient cases (%)	3 (6.7%)
Female patients that have given birth	0
Patients born with meconium ileus (%)	7 (15.5%)
Patients that have undergone lung transplantation	0

BMI—body mass index, FEV1—forced expiratory volume in one second; CF—cystic fibrosis.

## Data Availability

Not applicable.

## Supplementary Materials

The following supporting information can be downloaded at: https://www.mdpi.com/article/10.3390/diagnostics12112893/s1, Table S1: Genotypes of Latvian CF patients diagnosed between 1997 and 2022 and information about these genotypes held in the CFTR2 database (last updated 29 April 2022).
