# Supplementary material for: Clinical and Genetic Characterisation of Cystic Fibrosis Patients in Latvia: A Twenty-Five-Year Experience
Source: Diagnostics (Basel). 2022 Nov 21;12(11):2893. doi: 10.3390/diagnostics12112893 (PMC9689702; doi:10.3390/diagnostics12112893)
Supplement: Supplementary file 1 [file diagnostics-12-02893-s001.zip › diagnostics-2021488-supplementary.pdf]

diagnostics

Supplement

Table S1. Genotypes of Latvian CF patients diagnosed between 1997 and 2022 and information about these genotypes held in the CFTR2 database (last updated April 29, 2022).

Genotype according to traditional nomenclature	1st allele according to HGVS	2nd allele according to HGVS	N	%	Genotype described in n individuals cftr2.org database	Pancreatic function reported cftr2.org database[a]
F508del/F508del	c.1521_1523delCTT p.(Phe508del)	c.1521_1523delCTT p.(Phe508del)	34	48.5	33,984	PI (2%PS)
F508del/3849+10kb C>T	c.1521_1523delCTT p.(Phe508del)	c.3718-2477C>T p.(?)	2	2.8	260	PS (35%PI)
F508del/W1282R	c.1521_1523delCTT p.(Phe508del)	c.3844T>C p.(Trp1282Arg)	1 (2 sibs)	2.8	Second variants not reported in cftr2.org	NA
F508del/W1282X	c.1521_1523delCTT p.(Phe508del)	c.3846G>A p.(Trp1282Ter)	1	1.4	770	PI(2%PS)
F508del/R1066H	c.1521_1523delCTT p.(Phe508del)	c.3197G>A p.(Arg1066His)	3	4.2	56	PS(31%PI)
F508del/2143delT	c.1521_1523delCTT p.(Phe508del)	c.2012delT p.(Leu671Ter)	1	1.4	66	PI(6%PS)
F508del/dele 2,3	c.1521_1523delCTT p.(Phe508del)	c.54-5940_273+10250del21kb p.(Ser18ArgfsX16)	2	2.8	260	PI(0%PS)
F508del/W57R	c.1521_1523delCTT p.(Phe508del)	c.169T>C p.(Trp57Arg)	2	2.8	Second allele not reported	
F508del/394delTT	c.1521_1523delCTT p.(Phe508del)	c.262_263delTT p.(Leu88IlefsX22)	1	1.4	209	PI(2%PS)
F508del/2184insA	c.1521_1523delCTT p.(Phe508del)	c.2052dupA p.(Gln685ThrfsX4)	1	1.4	220	PI(13%PS)
F508del/ CFTRdup6b-10	c.1521_1523delCTT p.(Phe508del)	c.(743+1_744-1)_(1584+1_1585-1)dup p.(?)	1 (2 sibs)	2.8	13	PI(0%PS)
F508del/del6-10	c.1521_1523delCTT p.(Phe508del)	c.(743+1_744-1)_(1584+1_1585-1)del p.(?)	1 (2 sibs)	2.8	Second allele not reported	
F508del/L1335Pro	c.1521_1523delCTT p.(Phe508del)	c.4004T>C p.(Leu1335Pro)	2	2.8	11	55% PI/45%PS
F508del/L138ins	c.1521_1523delCTT p.(Phe508del)	c.413_415dupTAC p.(Leu138dup)	1	1.4	Not reported genotype	
F508del/621+1G>T	c.1521_1523delCTT p.(Phe508del)	c.489+1G>T p.(?)	1	1.4	884	PI
F508del/R334W	c.1521_1523delCTT p.(Phe508del)	c.1001G>A p.Arg334Gln	1	1.4	231	PS

F508del/A455E	c.1521_1523delCTT p.(Phe508del)	c.1364C>A p.Ala455Glu	1	1.4	355	PS (33% PI)
W1282R/2143delT	c.3844T>C p.Trp1282Arg	c.2012delT p.Leu671Ter	1	1.4	Not reported second allele	Pancreas function[a]
Dele2,3/dele2,3	c.54-5940_273+10250del21kb p.(Ser18ArgfsX16)	c.54-5940_273+10250del21kb p.(Ser18ArgfsX16)	1	1.4	17	PI (6%PS)
1677delTA/1677delTA	c.1545_1546delTA p.(Tyr515ter)	c.1545_1546delTA p.(Tyr515ter)	1	1.4	32	PI (0%PS)
Dele2,3/P67L	c.54-5940_273+10250del21kb p.Ser18ArgfsX16	c.200C>T p.Pro67Leu	1	1.4	Not reported genotype	
W1282X/R553X	c.3846G>A p.Trp1282X	c.1657C>T p.Arg553X	1 (2 sibs)	2.8	7	PI (14%PS)
Ser168Ter/Leu1335Pro	c.503C>A p.Ser168Ter	c.4004T>C p.Leu1335Pro	1	1.4	Second allele not reported	
F508del/NA	c.1521_1523delCTT p.(Phe508del)	-	3	4.2	NA	
R553X/NA	c.1657C>T p.Arg553X	=	1	1.4	NA	

[a]Pancreas function data gained from the cftr2.org database from reported patients; PI – pancreas insufficient, PS – pancreas sufficient, NA – not available or detected.
